# Gathering speed and countering tensions in the rapid learning health system

**DOI:** 10.1002/lrh2.10358

**Published:** 2023-01-03

**Authors:** Robert J. Reid, Sarah M. Greene

**Affiliations:** ^1^ Institute for Better Health, Trillium Health Partners Mississauga Ontario Canada; ^2^ Dalla Lana School of Public Health University of Toronto Toronto Ontario Canada; ^3^ National Academy of Medicine Washington District of Columbia USA

**Keywords:** collaboration, health care economics and organizations, health system, learning health system

## Abstract

The vision of the learning health system (LHS), conceptualized 15 years ago, is for the rapid generation, use, and spread of high‐quality evidence that yields better health experiences, outcomes, efficiencies, and equity in everyday practice settings across communities. However, despite the emergence of many useful LHS frameworks and examples to guide adoption, large gaps remain in the speed and consistency with which evidence is generated and used across the range of settings from the bedside to the policy table. Gaps in progress are not surprising, however, given the tensions that predictably arise when key stakeholders—researchers, health systems, and funders—comingle in these efforts. This commentary examines eight core tensions that naturally arise and offers practical actions that stakeholders can take to address these tensions and speed LHS adoption. The urgency for attenuating these tensions and accelerating health system improvements has never been higher. Timeliness, rigor, and prioritization can be aligned across stakeholders, but only if all partners are intentional about the operational and cultural challenges that exist.

The sluggishness and inconsistency with which high‐quality evidence is created, taken up, and spread to achieve benefit across populations continue to frustrate policymakers, government payers, clinicians, and patients.^1^ COVID‐19 has shone a bright spotlight on the evidence gaps occurring from the bedside to the policy table and the need for locally meaningful, high‐value evidence under very short timelines.^2^ The learning health system (LHS), conceived in 2007,^3^, ^4^ envisions a union of care delivery and research enterprises with a goal of rapidly generating and using evidence to improve health, care experiences, efficiencies, and equity within and across populations.^5^

Many useful LHS conceptual frameworks, lexicons, logic models, taxonomies, and examples^3^, ^5^, ^6^, ^7^, ^8^, ^9^, ^10^, ^11^, ^12^, ^13^ have emerged, all built on learning cycles where data are generated in usual care settings, knowledge is rapidly generated from these data and then widely used, refined, and scaled to advance policy and practice.^10^, ^12^, ^14^ While there is scant disagreement with the LHS ideal, a recent Global Evidence Commission report^2^ signals that the LHS vision remains unfulfilled across the world from its inception 15 years ago. Health research remains largely disconnected from health systems; deficiencies persist in the creation of intermediary evidence generation, implementation, and learning supports. While disappointing, these conclusions are not surprising given the real‐world tensions that often impede—singly or in combination—the rapid learning process. Recently conducted scoping and other reviews have articulated the range of organizational and sociocultural complexities and challenges^15^, ^16^, ^17^, ^18^ that may be eased or aggravated by financial, technical, or operational barriers to change. Because health systems are not built de novo, adding new features to health systems also requires that old structures and practices, often tenaciously embraced, be de‐implemented and discarded. Based on these reviews, this commentary outlines eight core tensions that predictably arise, and offers practical takeaways for researchers, health systems, and funders to counter these tensions.

## 1 AGREEING ON PURPOSE: MEETING BUSINESS OBJECTIVES OR PRODUCING GENERALIZABLE EVIDENCE?

Because different stakeholders often hold different perspectives, agreeing on the overall goals of learning is fundamental^6^, ^8^, ^16^, ^17^ but may be neglected. From the perspective of health system *leaders*, the creation and use of rapid‐cycle evidence to guide change is most valuable if it helps them attain business or performance objectives, such as improving the experience, reducing clinical variation, or gaining market share. Such objectives are typically set by senior executives or government officials and are often confined to short‐term planning, business, or electoral cycles. For system leaders, learning objectives that are longer term and reach across systems are often less attractive because they can lose immediate relevance and local nuance, and may raise concerns about reputational risk and competitiveness. This is particularly the case when systems operate in the same local market and compete for talent, reputation, and scarce resources.

In contrast, health system *researchers* are motivated by creating generalizable knowledge; local improvement may be one objective buried among many. Often driven by the demands of research funders, local distinctions often become variables for adjustment rather than prime motivations. Moreover, learning activities often persist across multiple business cycles by design and are not aligned with the short windows of opportunity when business decisions are made.

Health system researchers can address this tension at the outset by gaining agreement among stakeholders that learning activities will produce *both* generalizable evidence and address performance improvement priorities in realistic timeframes. “Boundary spanners” who serve dual roles as both health system leaders and researchers can facilitate agreement on shared purpose and address practical issues. Healthcare system learning networks^19^, ^20^, ^21^ exemplify our ability to navigate this tension, owing to their commitment to build trusted internal partnerships between research and care delivery, and align values, problems, and business objectives with embedded research and development enterprises. These networks allow system leaders and researchers to identify affinities, accelerate learning with the use of common data infrastructures, shared ethical review boards,^22^ and integrated knowledge translation platforms and other aligned resources.

## 2 BROADENING THE LEARNING GOALS: HEALTHCARE SYSTEM OR HEALTH SYSTEM?

The concept of a *learning healthcare system* has evolved to that of the learning health system, given that health is the overall product of interest, not just the production of quality healthcare.^23^ Receipt of healthcare is only one of numerous factors that determine individual and population health. Numerous social drivers are equally or more influential.^24^ Yet, a clear slate of concrete actions healthcare systems could or should take to broadly affect these underlying drivers is in formative stages at best. In this broader conceptualization, the LHS is less about closing gaps in the application of existing evidence, and more about building the core evidence base of how healthcare systems, whose accountabilities are for delivering care services, can effectively influence social drivers of wellbeing.

Consideration of these wider determinants also necessitates the expansion of system partnerships beyond healthcare to other sectors with health impact (e.g., public health, education, and housing).^25^ Both health systems and researchers are now partnering with community‐based organizations^13^ as a means of addressing these exogenous factors that are apart from the healthcare experience. However, this introduces considerable complexity. Tension arises when social service providers and community‐based organizations have different objectives, accountabilities, frameworks, and partners, obliging healthcare systems and researchers to thoughtfully navigate these new complexities and intricate relationships. The fragmentation of healthcare simply creates an uphill climb, whether the goal is to address social drivers and equity, or clinical drivers.

Because of these complexities, the LHS movement has mostly remained narrowly focused on healthcare interventions. However, national research funders are now prioritizing innovations in health service delivery that address socioeconomic issues and improve health equity.^26^, ^27^, ^28^ Examples of this type of applied research are growing in areas including food insecurity,^29^ housing instability,^30^, ^31^ and others.^32^, ^33^, ^34^, ^35^, ^36^, ^37^ Encouragingly, recognition that health is more than healthcare is spurring innovative thinking about testing new integrated models of service provision that are tailored to specific under‐resourced populations. Exemplars include the Integrating Care for Kids initiative,^38^ Kaiser Permanente's Social Needs Network for Evaluation and Translation,^39^ and the Social Care Innovation Network.^40^

## 3 SETTING THE LEARNING AGENDA: RESEARCH, HEALTH SYSTEM, OR COMMUNITY PRIORITIES?

Traditional discovery science prioritizes investigator‐led research where funding proposals are chosen according to their scientific significance, investigator expertise, and the potential scientific advancement from generalizable new knowledge or capabilities produced. In the applied sciences that encompass LHS such as implementation science and applied clinical informatics, priorities shift to local issues faced by health service organizations. A tension, then, is how to broaden the aperture of research funding and capacity‐building priorities that are inclusive of the local system and community priorities,^6^ many of which are dynamic and specific in nature. For instance, health systems may prioritize cancer screening rates for their patient population in response to government quality indicators, whereas communities may want to focus on upstream needs such as building mental health resilience in schools. When research significance, health system, and communities' priorities and capacities align, LHS programs are likely to thrive. However, formal mechanisms to align priorities, particularly with broader health sector partners, patients, and communities are often absent. Helpfully, research funders are increasingly bringing health systems and community representatives into the prioritization and funding decision‐making process. For instance, funders are now incorporating input from health system decision makers in setting funding priorities, requiring coequal leadership by patient/community partners, and creating new opportunities for research directed at local system needs.^41^, ^42^, ^43^, ^44^ The Patient‐Centered Outcomes Research Institute (PCORI) also seeks to alleviate this tension by providing public‐facing plain‐language summaries of research findings as a means of promoting uptake and increasing reach.^45^

## 4 SPEEDING THE APPROACH: METHODOLOGICAL PURITY OR OPERATIONAL PRACTICALITY?

High‐quality, rigorous research often takes considerable time and effort to design, conduct and disseminate. However, the pace of decision‐making in many health systems cannot await the long horizons of a multi‐year randomized trial or another longitudinal study. While randomized trials are considered the gold standard in clinical research, the design features (randomization, masking, intervention fidelity, clinic burden, and extended follow‐up) are cumbersome in many health systems and may be undesirable in settings where agility and responsiveness are key. Instead, health systems' available data on their populations are often seen as “good enough” for guiding time‐sensitive decisions about program design, staffing mix, or financing arrangements. However, without sufficient attention on methods, these data and their perceived meanings can be seriously flawed, resulting in poorly conceived interventions with unintended consequences.

To address the tensions of rigor and rapidity concomitantly, scientists are applying pragmatic trial designs^46^, ^47^, ^48^, ^49^ with novel statistical methods,^50^ living evidence syntheses,^51^, ^52^ rapid qualitative thematic analyses,^53^, ^54^, ^55^ indigenous ways of knowing,^56^ and other complementary and advanced methods. Another important tactic is to leverage vast stores of electronic health data for secondary analyses, equity‐sensitive predictive modeling, or real‐time research which could take the form of rapid prototyping, ethnographic studies, synthetic research,^57^ and other rigorous time‐sensitive designs. Hence, while traditional clinical trials still have an essential role in intervention comparisons, the heterogeneity and complexity of today's healthcare demand expanded thinking about pragmatic study designs that can deliver reliable and ready insights to support change.

## 5 DATA FOR LEARNING: PRIMARY DATA COLLECTION OR SECONDARY USE?

Clinical and health system operational data are the lifeblood of the LHS. The richness, volume, coverage, and complexity of structured and unstructured health data provide tremendous opportunities for learning but produce tensions when data infrastructures are simultaneously used for care, business functions, quality improvement (QI), and research.^6^, ^7^, ^16^, ^17^, ^18^ Operational applications of data can tolerate some messiness but variation in the provenance, availability, latency, and quality of data collected at the point of care collide with the researcher's need for standardization, curation, and highly reliable and complete source data. Another tension germane to the LHS and data is the ability of a health system to “serve up” data‐driven and high‐quality research evidence at the right moment, usable in practice for real‐time clinical or managerial decision‐making. The primary purpose of today's electronic health record (EHR) is for documentation and billing—enabling the EHR to ingest information that a clinician can utilize during a patient encounter may be a lower priority of the health system's information technology decision‐makers. Health systems typically deploy enterprise data warehouses to store and analyze many petabytes of data, learning activities may be hampered if they do not longitudinally connect clinical, administrative, and patient‐reported data at the level of the individual and across defined populations. In particular, collecting high‐quality patient‐reported outcomes and experience data are of emerging importance in LHS research, but pose significant challenges for health systems to mount efforts for systematic and wide‐scale collection at the point of care. Several solutions are emerging, namely creating C‐suite positions overseeing health data, creating “communities of analysts” who work at the research and clinical operations interface, and developing robust, responsive data governance approaches that include patient, community, and equity‐related perspectives.

## 6 ENSURING ETHICAL OVERSIGHT: RESEARCH OR QUALITY IMPROVEMENT?

Clinical practice and research are codified as unique and independent enterprises guided by distinct ethical frameworks.^58^ For clinical care, healthcare professionals and organizations have an overriding ethical responsibility to serve the best interests of their patients and populations. This responsibility also extends to the imperative for QI and the use of data at the point of care for continual learning.^59^ For research, where the main purpose is to produce generalizable new knowledge and not directly improve care, researchers must uphold the Belmont principles including informing individuals of research activities, minimizing risks, protecting confidentiality and privacy, and allowing informed choice about participation.

Because LHS activities often have the dual purposes of improving care locally and producing new insights relevant to other systems, ethical oversight falls in the fuzzy boundary between ethics of research, overseen by research ethics boards, and that of clinical care and QI, the purview of clinical oversight mechanisms. LHS scientists are obliged to seek review from research ethics boards for learning initiatives that are explicitly designed to improve care locally and to derive broad inferences. While this may seem sensible, solely applying a research ethics lens to these endeavors can have the perverse, if unintended consequence of dampening down the ethical imperative to directly improve care locally. The transactional costs of research ethics review, even for minimal‐risk studies, are a distinct disincentive to engage in LHS activities that are deemed research, particularly when the risk is relatively low.^59^ As well, requirements for approved fixed protocols that can only be modified with amendments are antithetical to rapid LHS approaches characterized by frequent adjustments to interventions based on derived knowledge. Finally, the duty to inform patients of embedded research that is a regular part of their care, such as a cluster randomized trial of alternative hospital discharge processes, presents another ethical conundrum. Strategies to navigate this tension include merging clinical and research oversight functions within systems,^60^ adoption of tools that attempt to disentangle QI from research (such as the ARRECI tool^61^), the use of general opt‐out consent processes or master protocols,^62^ and adoption of new disclosure mechanisms for LHS activities.^60^, ^63^, ^64^, ^65^

## 7 AIMING FOR SUSTAINABILITY: SHORT‐TERM PROJECTS OR ONGOING INFRASTRUCTURES?

Grant‐funded research, by nature, is project based and time limited. Similarly, health system initiatives have defined life cycles nested within organizational business plans. Some funding agencies have developed sustainable infrastructures intended to serve as scaffolding for a long‐term research agenda^19^, ^21^, ^66^, ^67^, ^68^, ^69^ that facilitates dissemination and implementation of successful research into real‐world practice and builds over time. Typically though, funding is granted to demonstrate the efficacy of an intervention or program, with fewer opportunities to continue with implementation, spread, and sustainability. Similarly, in health systems, earmarked projects may be prioritized for a budget cycle or two, then supplanted by other emergent priorities. However, sustainability cannot be an afterthought. From the outset, researchers need to consider the contextual factors that can support or impede the uptake of successful interventions.^70^, ^71^ Thinking through—and documenting—the financial, technical, political, and sociocultural barriers and facilitators as interventions are deployed can support eventual scale‐up and accelerated learning. The creation of sustainable infrastructures ensure that scientists and health system operators can respond to longer term and evolving needs.^72^, ^73^, ^74^

## 8 APPLYING TECHNOLOGY: SUPPORTING IMPROVEMENT OR RAPID DISRUPTION?

Health service delivery is a fertile space for quick‐cycle, technology‐driven innovations that span the care continuum including mobile health apps that support health behavior or symptom management, sophisticated devices that improve diagnostic or therapeutic accuracy, cloud computing to support large‐scale data mining and machine learning, and infrastructure developments that support new care delivery models (e.g., telehealth platforms).^75^ The thirst for rapid advances in medical care may meet requirements for patient codesign and systematic evaluation, and the risks of bringing technology‐driven solutions to market in a ubiquitous manner. The LHS is intended to be an adaptive organism, and embracing new technologies for learning at speed is inherently a positive attribute. Nonetheless, healthcare operational leaders and decision‐makers can benefit from partnerships with researchers to evaluate the clinical and managerial utility, equity effects, inherent risks, and relative improvement of such technologies to proceed with the adoption, spread, and necessary adaptations with greater confidence. Increasingly, LHS researchers are honing a range of relevant competencies in data science, engagement, improvement, equity, and implementation science^76^, ^77^ and can be full partners to health systems in the technology assessment process and simultaneously generate critical new evidence.

## 9 PRACTICAL STEPS FOR RESEARCHERS, SYSTEM LEADERS, AND FUNDERS TO LESSEN LEARNING HEALTH SYSTEM TENSIONS

Consistently mobilizing the best, latest evidence and applying it forward so that it reaches every person in every encounter is central to the realization of a true LHS. The time frame of last 15 years has grounded us in LHS concepts and methods, and the barriers to progress have been well articulated.^16^, ^18^ Focused attention on practical actions that stakeholders can take is the newest imperative to speed LHS adoption and gain accelerated momentum and consistency. Based on the tensions described herein, Table 1 distills several practical actions for key stakeholders—health system leaders, scientists, and research funders. As stakeholders consider and apply these actions, it is important to apply a learning mindset recognizing potential secondary tensions that might arise and developing mitigation strategies.

**TABLE 1:** Stakeholder Actions to Reduce Learning Health System (LHS) Tensions

LHS Scientists
Understand health systems' strategic plan, key business priorities, and capacities for change Identify opportunities within organizational business plans to apply rigorous and rapid analytic approaches for change Consider practical multimethod applications and be sensitive to the need for adaptation over time Value internal expertise of system and community leaders and practitioners Apply a “servant‐leader” mindset to address health system challenges that might depart from investigator priorities
Health System Leaders
Establish mechanisms to set iterative learning priorities with researchers, communities, patients, and families Complement systems business intelligence, decision support, and QI teams with health system scientists Build data and technology platforms, governance, and safeguards that can jointly accommodate care, improvement, and research needs Embrace advanced research methods to rapidly understand issues, contextualize existing evidence, codesign solutions, understand change results, and adapt and spread learnings Leverage research on strategies and methods for patient/community engagement and codesign Ensure that internal ethics review systems bridge research, QI, and innovation
Research Funders
Build research career development programs for embedded LHS scientists, especially among traditionally underrepresented groups Develop research award programs that align with national and system‐level health improvement priorities focused on the “how” of change rather than the “what” Provide tangible and sustainable support for research and improvement programs that involve patient‐ and community‐level partnerships and coproduction Fund research on novel methods that enable rapid design, prototyping, and implementation, with emphasis on multimethod and practical approaches Value research that results not only the production of generalizable knowledge but also in local improvement and health system business success Incentivize research that has a demonstrable impact on healthcare outcomes, and de‐emphasize investigator‐initiated research that might only yield incremental change

Abbreviation: QI, quality improvement.

The immensity of the research landscape and the complexity of both health and healthcare create significant impediments to generating and harnessing robust evidence at scale. However, the urgency has never been higher—health gains are slowing or receding, healthcare costs increasing, equity gaps widening, and the return on investment in clinical research is not fully realized. Identifying and attenuating the key tensions that can arise when researchers, health systems, and communities come together to apply a learning mindset could accelerate our ability to harness evidence to improve population health, equity, experiences, and costs. Timeliness, rigor, and prioritization can be aligned across stakeholders; however, only if all partners are intentional about the operational and cultural challenges of closing the gaps from data to knowledge to impact.
